# Evaluation of Folate-Functionalized Nanoparticle Drug Delivery Systems—Effectiveness and Concerns

**DOI:** 10.3390/biomedicines11072080

**Published:** 2023-07-24

**Authors:** Muhammad Aiman Irfan Ibrahim, Rozana Othman, Chin Fei Chee, Faisalina Ahmad Fisol

**Affiliations:** 1 Department of Pharmaceutical Chemistry, Faculty of Pharmacy, Universiti Malaya, Kuala Lumpur 50603, Malaysia; aimanedogawa4979@gmail.com; 2 Centre for Natural Products Research & Drug Discovery (CENAR), Universiti Malaya, Kuala Lumpur 50603, Malaysia; 3 Nanotechnology & Catalysis Research Centre (Nanocat), Universiti Malaya, Kuala Lumpur 50603, Malaysia; cheechinfei@um.edu.my; 4 Malaysian Institute of Pharmaceuticals and Nutraceuticals (IPHARM), National Institutes of Biotechnology Malaysia (NIBM), Gelugor 11700, Malaysia; faisalinafisol@gmail.com

**Keywords:** folate receptors, folate receptor-targeting, folic acid, low tumor selectivity, nanoparticle drug delivery systems

## Abstract

Targeting folate receptors is a potential solution to low tumor selectivity concerning conventional chemotherapeutics. Apart from antibody–drug conjugates, folate-functionalized nanoparticle drug delivery systems are interesting to be explored due to many advantages, yet currently, none seems to enter the clinical trials. Multiple in vitro evidence is available to support its efficacy compared to the non-targeting carrier and free drug formulation. Additionally, several studies pointed out factors affecting its effectiveness, including surface properties and endosomal trapping. However, in vivo biodistribution studies revealed issues that may arise from folate receptor targeting, including rapid liver uptake, subsequently reducing the nanoparticles’ tumor uptake. This issue may be due to the folate receptor β expressed by the activated macrophages in the liver; route of administration and tumor location might also influence the targeting effectiveness. Moreover, it is perplexing to generalize nanoparticles reported from various publications, primarily due to the different formulations, lack of characterization, and experimental settings, making it harder to determine the accurate factor influencing targeting effectiveness.

## 1. Introduction

For conventional or early-generation chemotherapeutic agents (for example, paclitaxel, docetaxel, and doxorubicin), selectivity is a concerning issue, given they are non-specifically distributed in the body, affecting both normal and tumor cells during the treatment. Hence, chemotherapeutics that selectively target tumors are explored to overcome this systemic toxicity problem, hoping to improve patients’ survival time and quality of life. Generally, tumor-targeting involves utilizing the tumors’ vascular abnormalities. The targeting strategy is classified into passive and active targeting. Passive targeting involves transporting nanoparticle drug delivery systems through the leaky tumor environment by passive diffusion or convection. In active targeting, one or different targeting ligands are attached to the nanocarrier surface or covalently linked to a therapeutic agent via a cleavable spacer for binding non-covalently to the receptors overexpressed by cancers or receptors not expressed by normal cells [1,2].

**Figure 1:**
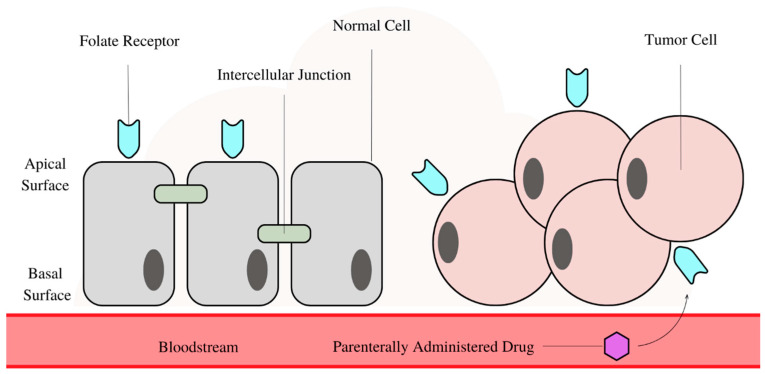
A simple diagram illustrating the folate receptor (FR) position on the normal cell apical surface compared to the FR randomly distributed over the tumor cell surface. With the intercellular junction in place, the drug is incapable to interact with FR on the apical surface.

One particular target gaining significant interest is the folate receptor (FR). Currently, there are four FR isoforms identified: α, β, γ, and δ in which FRα is the most investigated. It is typically expressed on the apical surfaces of normal epithelial cells in the choroid plexus, lungs, mammary duct, and kidneys. Due to its apical location away from the bloodstream, it is isolated from parenterally administered drugs. Nonetheless, the apically restricted FR is redistributed over the tumor cell surface due to the intercellular junctions collapse from carcinogenesis. Hence, the parenterally administered drugs selectively interact with FRs present in malignant tumors instead of with those from normal cells, as illustrated in Figure 1 below. Additionally, FRα is often overexpressed in tumor cells, increasing the probability of the ligand binding to the tumor instead. These two factors contribute to the selectivity of the FR targeting strategy. The tumors’ overexpression of FRs is possibly due to their increasing demand for folic acid, essential for cell maintenance and proliferation. Folic acid act as a one-carbon donor in the biosynthesis of purines and thymidylate, crucial for the de novo synthesis of RNA and DNA. Additionally, folate is essential for the cobalamin-dependent synthesis of methionine and methylation of DNA, lipids, histones, and neurotransmitters [2].

## 2. Current Updates on the Clinical Trials

In the past, the folate-targeting approach was explored right after the report by Bart Kamen’s group (the University of Texas Southwestern Medical Centre) which stated that folates enter the cells via a receptor-mediated endocytic process [3,4]. Some early attempts included using the nitro heterocyclic bis(haloethyl)phosphoramidite conjugates (research using this class of conjugates has been abandoned), platin conjugate, FdUMP conjugate, Taxol conjugate, and maytansine conjugate [3].

Currently, several FR-targeted therapeutics are undergoing clinical trials, including MORAb-202. It is an antibody–drug conjugate (ADC) consisting of farletuzumab (FRα-binding antibody) and eribulin (a microtubule-targeting agent) linked by a cathepsin-B cleavable linker [5,6]. The recent first-in-human phase 1 study for patients with advanced solid tumors (triple-negative breast cancer, ovarian, endometrial, and non-small cell lung cancer) demonstrated that MORAb-202 was well-tolerated at 0.3–1.2 mg/kg doses (yield 0.2–0.85 mg/m^2^ eribulin) for every three weeks, lower than the approved dose of eribulin mesylate: 1.4 mg/m^2^ or equivalent to 1.23 mg/m^2^ eribulin on days 1 and 8 of a 21-day cycle. Two from 22 patients experienced increasing alanine aminotransferase and γ-glutamyl transferase (grade 3 treatment-emergent adverse events, TEAE). There was no case of grade 3 bone marrow suppression, which indicated a mild safety profile. However, no maximum tolerated dose (MTD) was established in this dose-escalation study [6], prompting more investigation to further construct its safety profile.

Compared to mirvetuximab soravtansine (IMGN853), an ADC consists of a humanized anti-FRα monoclonal antibody (M9346A) conjugated to the DM4; a cytotoxic maytansinoid effector molecule [7,8], MORAb-202, uncommonly caused eye disorders of grade 1 (two out of twenty-two patients) [6]. However, this may be because of the tiny patient samples in this study or the different payloads used (i.e., the cytotoxic component of the ADC). In the phase 1 dose-escalation study of IMGN853 in patients with FRα-positive solid tumors, ocular TEAEs of grades 1 and 2, including reversible blurred vision or/and keratopathy, were commonly reported. The molecular mechanisms fundamental to these observations remain imperfectly elucidated despite these issues reported for numerous ADCs using various cytotoxic payloads to target different antigens. One proposed reason for this event is that prolonged retention in the circulation contributed by the stable linker is adequate to magnify overall exposure in normal tissues [7].

In the phase 1 expansion study of IMGN853 in the fallopian tube, platinum-resistant ovarian, or primary peritoneal cancer, and similar grades 1 and 2 mild adverse events were reported, including ocular disorders at a relatively high frequency. Proactive measures, including daily lubricating eyedrops, sunglasses in daylight, and avoiding contact lenses, help to manage the issue. The therapeutic seems promising in patients with platinum-resistant ovarian cancer receiving fewer lines of prior therapy. However, responding patients eventually experienced inherent and acquired resistance, which the mechanism remains to be defined. The combination therapy using molecularly targeted therapeutics with nonoverlapping toxicities and different mechanisms of action is essential in managing epithelial ovarian cancer (EOC) to delay or counteract IMGN853 resistance [8].

A recently published phase 1b study of IMGN853 in conjunction with bevacizumab in platinum-resistant ovarian cancer patients observed no new safety signs nor any clinically significant enhancement of the forecasted toxicities for both therapeutics. None of the sixty-six enrolled patients experienced any grade 3 or higher TEAEs, and two patients were discontinued because of persistent grade 2 peripheral neuropathy. All hypertension cases related to bevacizumab were easily managed, with none causing discontinuation [9]. The phase 1b escalation study of IMGN853 in combination with carboplatin in platinum-sensitive ovarian cancer patients reported a similar outcome in which no new safety signals appeared, and the toxicities detected were anticipated based on the established profiles of each therapeutic. The combination used demonstrated comparable mitigation in moderate to severe hematological toxicities than reported for the carboplatin/paclitaxel combination, despite the rising frequency of grade 3 events [10].

Regardless of these outcomes from the clinical trials, ADC is associated with other limitations, including difficulties in penetrating deeply into the solid tumor because of the large size of antibodies, immunogenic potential, the possibility of premature drug releases due to their long circulation period, and expensive production of ADCs. The small molecule-drug conjugate is acknowledged as a possible solution to the issues above. Vintafolide (EC145), a folic acid–vinca alkaloid desacetylvinblastine hydrazide (DAVLBH) conjugate, seemed promising when combined with docetaxel in NSCLC patients. However, a phase III trial evaluating EC145/doxorubicin hydrochloride liposome in platinum-resistant ovarian cancer patients had been suspended in 2014, perhaps due to the failure to enhance progression-free survival [2]. Currently, there is no recent update on the development of Vintafolide.

There are other FR-targeting therapeutics under development and in clinical trials, for example, etarfolatide (EC20), EC17, and OTL38. Nonetheless, apparently, no folate- functionalized nanoparticle drug delivery system has successfully completed any clinical trial stages at the moment, despite numerous having been successfully developed and demonstrating its efficacy in pre-clinical stages. It is intriguing to investigate if any obstacles hinder the progression of folate-functionalized nanoparticle drug delivery systems in clinical trials. The nanoparticle drug delivery system has many advantages, including therapeutic payload compartmentalization from the surrounding normal tissue while in blood circulation. Targeting ligand-functionalized nanocarriers is particularly useful for conventional chemotherapeutic payloads with low tumor selectivity. Therefore, this review aims to evaluate recently published pre-clinical studies on folate-functionalized nanoparticle drug delivery systems, which emphasize their effectiveness and any concerns that arise in the investigation.

## 3. Methodology

**Figure 2:**
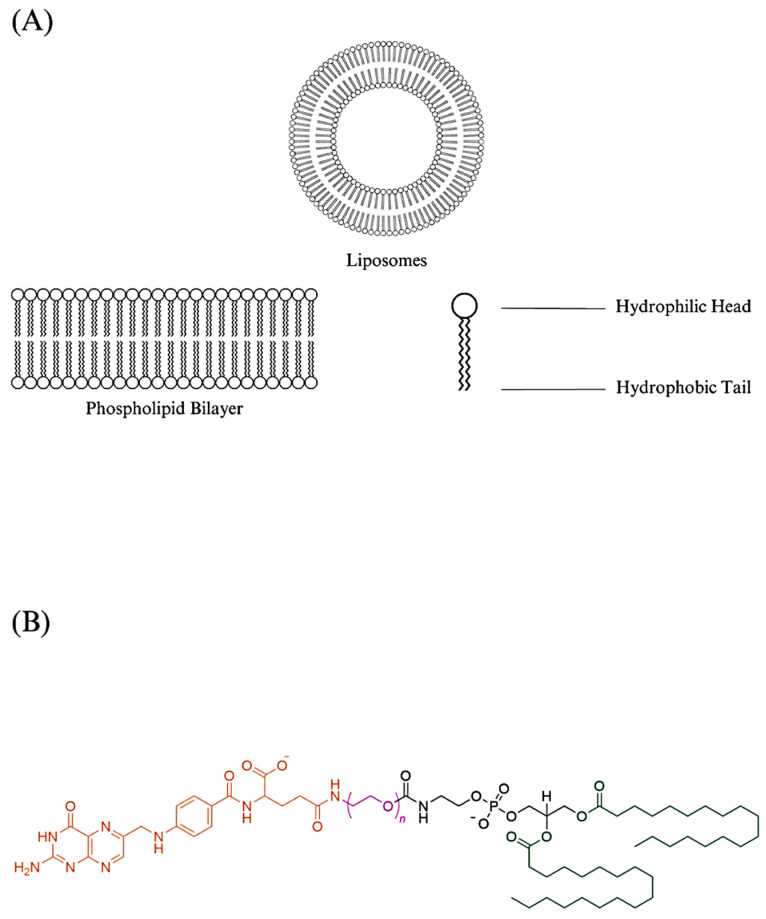
A simple diagram illustrating the structure of liposomes: (**A**) The phospholipid bilayer forms the liposomes’ shell; (**B**) Folate-DSPE-PEG. It is a typical component used in formulating folate-targeted liposomes [17,22,25]. The hydrophobic tails are highlighted in green. The hydrophilic PEG is highlighted in pink. The targeting folate is highlighted in orange.

**Figure 3:**
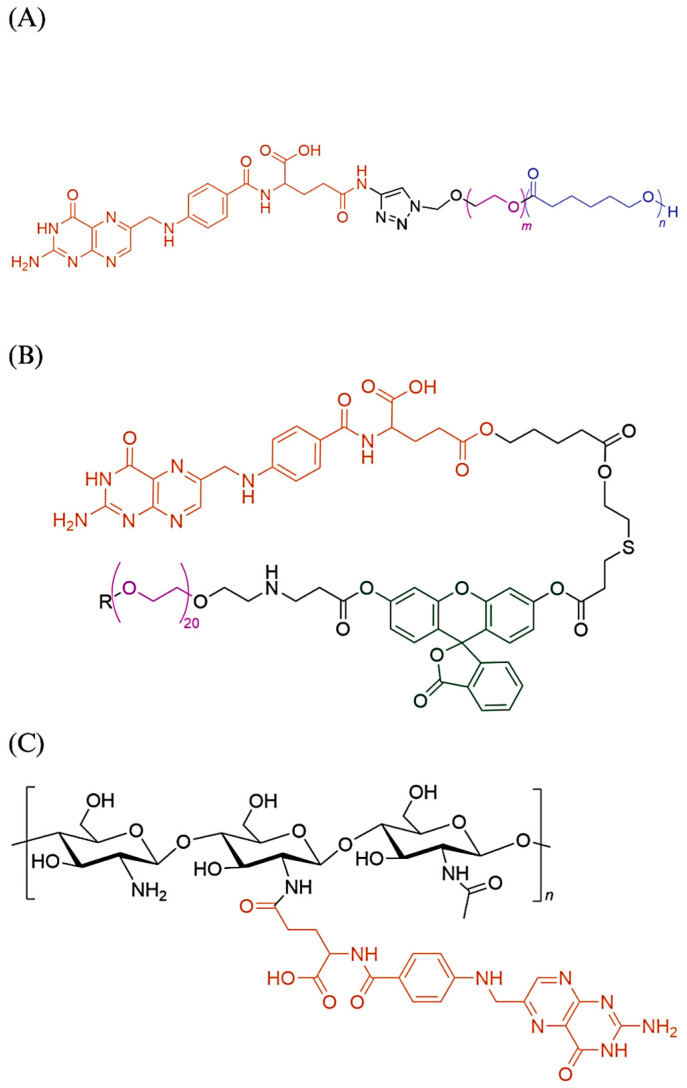
(**A**) Folate-PEG-Polycaprolactone [28]; (**B**) Folic acid and fluorescein isothiocyanate (FTIC) conjugated to a PEG core. R is equivalent to the other side of the PEG chain. FTIC is highlighted in green [21]. It is common to observe folic acid conjugated to PEG, either directly or through a linker; (**C**) Chitosan-Folate. The folic acid is conjugated to the chitosan deacetylated amine group along the chain. It is possible to conjugate other molecules to the amine group [20,23]. Another example of folate conjugated to the amine group from the source materials is folate conjugated to the branched polyethylenimine [19].

This review aims to evaluate the active targeting folate-functionalized nanoparticle drug delivery systems. PubMed was the primary database utilized to obtain the key papers used in the discussion. (“Folate Targeting” [Title/Abstract] OR “Folic Acid Targeting” [Title/Abstract]) AND (“Drug Delivery” [Title/Abstract] OR “Nanoparticles” [Title/Abstract]) were the keywords used in the search engine. The results (*n* = 131) were filtered to only show papers in the last five years (2018–2022, *n* = 57) since numerous papers have been published regarding this topic over the decades. Exclusion criteria applied to the results were: (1) Unavailable. (2) Review papers are excluded from the key papers. (3) Nanoparticles designed with multiple targeting strategies, including stimuli-sensitive and dual targeting agents. For the evaluation of folate-functionalized nanoparticles’ application beyond cancer, several non-cancer studies were included, given that the exclusion criteria are inapplicable to the study. This search strategy left 21 key papers which were used to aid the evaluation. The summary of key papers used to facilitate the discussion is presented in Table 1 below. Several nanoparticles’ structures from the papers listed are visualized in Figure 2 and Figure 3 below.

**Table 1:** Summary of key papers obtained from the search strategy presented above.

References	Nanoparticles’ Composition	Payload	Disease	Summary
[11]	Folate-coated PEG-conjugated graphene oxide	Protocatechuic acid	Hepatocellular carcinoma	Folate-coated nanoparticles demonstrated better anticancer activity than non-targeting nanoparticles and free protocatechuic acid in vitro.
[12]	Folate-coated PEG-conjugated graphene oxide	Protocatechuic acid and chlorogenic acid	Hepatocellular carcinoma	Folate-coated nanoparticles demonstrated better anticancer activity than non-targeting nanoparticles and free drugs in vitro.
[13]	Folate-conjugated single-wall carbon nanotube	*ITPA* siRNA	Cancers	The folate- conjugated single-wall carbon nanotube with an average length of >450 nm is better for FR-mediated endocytosis in vitro.
[14]	^64^Cu-radiolabeled folate-targeting liposomes	Copper chelator, DOTA	Cancers	Both tumor accumulation and circulation properties of liposomes may be lost due to functionalization.
[15]	Folate-functionalized mesoporous nanostructured silica systems	Triphenyl tin (IV) derivative	FR-overexpressed cancer cells	The nanoparticles are more active in targeting FR-overexpressed cancer cells when the quantity of functionalized folic acid is higher.
[16]	Folate-conjugated casein micelles	Monacan, Anka Flavin, and resveratrol	Breast cancer	The folate-conjugated micelle has similar cytotoxicity to the PEGylated phytozoa casein nanoparticle. Both nanoparticles have significantly higher cytotoxicity than free drugs. Significant reduction of body weight loss, tumor weight and volume, suppression of aromatase, NF-dB, VEGF, CD-1, and elevation of Caspase-3 demonstrated by both nanoparticles in vivo.
[17]	Folate-targeted liposomes composed of DOPE, cholesterol, DSPE-MPEG, and folate-peptide	Methotrexate	Arthritis	The folate-targeting liposomes have lower renal and hepatic elimination than the free methotrexate (MTX). The liposomes dosage equivalent to 2 mg/kg MTX, twice weekly, is similar to or better than 35 mg/kg MTX at reducing the swelling in the mice model.
[18]	Folate-targeting hyperbranched polyethylenimine-graft-polycaprolactone-block-PEG	siRNA	Ovarian cancer	The nanoparticles demonstrated excellent siRNA delivery profiles in vitro. The in vivo tumor uptake is affected by the route of administration. The intraperitoneal injection showed better tumor deposition over intravenous administration.
[19]	β-cyclodextrin and folic acid covalently conjugated to branched polyethylenimine	-	-	Developed new method for the nanoparticles’ synthesis. The FR-binding study using Lewis lung carcinoma suggests the conformation of the folate ligand on the surface is essential to maximize the binding.
[20]	Folate-chitosan-lipid conjugate	Cisplatin	Cancers	Significant increase in cytotoxicity, cell cycle arrest, and cellular uptake for folate-functionalized nanoparticles than the non-targeting nanoparticles.
[21]	Folic acid and fluorescein isothiocyanate conjugated to a PEG core	-	Hepatocellular carcinoma	The PEG enhanced the solubility of folic acid. The study suggests that intra-arterial administration is more efficient for targeted HCC detection than intravenous delivery.
[22]	Nanosuspension composed of DSPE-PEG-FA and soybean lecithin	Annonaceous acetogenins (ACGs)	Cancers	The folate-functionalized nanosuspension demonstrated significantly higher cytotoxicity against HeLa cells and better tumor growth inhibition in vivo than the non-targeting nanosuspension.
[23]	Folate-chitosan- selenium nanoparticles	FLuc mRNA	Cancers	Significant transgene expression for the FR-positive KB cells than other cells with little or negative FR.
[24]	Folate-functionalized PEG-modified PAMAM G4 dendrimers	5-fluorouracil (5-FU) and technetium-99 m (^99m^Tc)	Breast cancer	The nanoparticles are highly internalized by the 4 T1 cells than C2C12. The data suggest that PEG helps to reduce cytotoxicity. The complex significantly reduced the tumor volume in the mice model compared to the control group. The complex significantly accumulates in the liver and tumor tissue.
[25]	Folate-modified PEG liposomes, primarily using SPC, DC- Cholesterol, and DSPE-PEG	Oleuropein	Prostate cancer	The nanoparticles significantly inhibit cell viability more than plain oleuropein solution. The intravenous pharmacokinetic profile shows six times increase in AUC for nanoparticles than free oleuropein. The folate-modified nanoparticles increase weight loss resistance, tumor suppression, and survival probability in the mice.
[26]	Folate-functionalized pluronic micelles	Fisetin	Breast cancer	The folate-functionalized micelles demonstrated higher anticancer activity than non-targeting micelles and free fisetin in vitro against FR-overexpressed human breast cancer MCF-7 cell line.
[27]	Folate-functionalized PEG-coated liposomes	Fluorescent DiD/ ^3^H-cholesteryl hexadecyl ether/ Betamethasone	FR-positive immune cells in inflammatory diseases (e.g., colitis and atherosclerosis	The folate-targeted liposomes selectively bind to the FR-positive RAW 264.7 murine macrophage cell line in vitro and accumulate at inflammation sites in atherosclerosis and colitis murine models.
[28]	Folate-decorated poly(ε-caprolactone)-poly(ethylene glycol), PEG-PCL	-	-	PEG length particularly affects folate exposition and protein interaction. The nanoparticles with PEG length (2.0 kDa) are smaller than a shorter PEG length (1.0 kDa). The zeta potential is slightly negative, as typically exhibited by PEGylated nanoparticles. The characterization suggests the PEG moiety is in a mushroom conformation. The study suggests reduced nanoparticle uptake in human macrophages due to PEGylation. Significant uptake in FR+ KB cells than FR– A549.
[29]	Folic acid-dimethyl indole red (Dir)- bovine serum albumin (BSA)	siRNA	Cancers	Dir demonstrated selective noncovalent interaction with BSA than human serum albumin (HSA). The nanoparticles demonstrated fluorescent properties suitable for the targeted tumor cell imaging in vitro.
[30]	Folate-displaying exosome	siRNA	Cancers	Demonstrated that the delivery of the siRNA payload is unaffected by endosomal trapping.
[31]	Folate-functionalized PEGylated cyclodextrin	Docetaxel and siRNA	Colorectal cancer	The folate-functionalized nanoparticles demonstrated an enhanced apoptotic effect of docetaxel and downregulation of Re1A expression against CT26 cell lines and mouse model.

## 4. Discussion

### 4.1. Factors Affecting Folate-Functionalized Nanocarrier Effectiveness

Following a previous study by Conte et al. (2016) [32], Venuta et al. (2018) reported the conjugation of folic acid to the copolymer PEG-PCL hydroxyl-end moiety using the Click reaction (PEG length 1.5 kDa). The PEG moieties were presumed to form the nanoparticles’ outer corona while exposing the conjugated folate. However, the presence of serum proteins impacts the folate exposition in highly PEGylated micelle-like nanoparticles by forming a protein corona, shielding the folate from interacting with the FR. Hence, both studies introduced (2-hydroxypropyl)-β-cyclodextrin (HPβCD) to promote the PEG chain extension and folate exposition. Specifically, the key study investigated the formulation composed of non-targeting PEG-PCL (either 1.0 or 2.0 kDa PEG length), 20% by weight Fol-PEG-PCL described above, and HPβCD. Note that both studies employed different methods to prepare the nanoparticles despite similar formulation compositions. The motivation behind the inclusion of non-targeting nanoparticles into the formulation is unclear.

Further in vitro investigation into the interaction of the nanoparticles with human serum albumin (HSA) at physiologically relevant HSA concentration (500 μM) using fluorescence spectroscopy demonstrated that the nanoparticles’ surface (using 2.0 kDa PEG length) is fully occupied by HSA, forming a soft protein corona. The nanoparticles using 1.0 kDa PEG length remain relatively unaltered in terms of mean size. Presumably, more folate is exposed to the surrounding environment in a copolymer with 1.0 kDa PEG than 2.0 kDa PEG, impacting the active targeting. However, the study did not further validate this inference, preferably using an in vivo animal model. Interestingly, the surface PEG weight percentage for nanoparticles with shorter PEG is relatively higher than the longer PEG. The data suggest the protein corona is less likely influenced by the amount of PEG exposed on the surface; or, perhaps a threshold amount of surface PEG is required before the interaction with the protein becomes prevalent. Hence, further investigation will certainly help us to understand this problem better.

Based on the study using folate-modified PEG_5000_ liposome [25], in which the PEG length is significantly longer than used in previous studies, the biodistribution data from the excised tumor suggests enhanced targeting by the nanoparticles compared to the free drugs and non-targeting liposomes. Whether there is any significant protein interaction with the liposomes is unknown and impossible to postulate because there is no in-depth characterization of the nanoparticles’ surface, no protein interaction study conducted, and the nanoparticles’ composition used in this study and the previous one are different. In another study using folate-functionalized PEG-modified PAMAM G4 dendrimers [24], the study claimed the folate and PEG reduced the cytotoxicity effects of the dendrimers.

Unfavorable folate conformation on the surface of the nanoparticles could limit its interaction with the folate receptor. The study by Kasprzak et al. (2018) provides a novel synthesis method for nanocarriers consisting of both β-cyclodextrin and folic acid on the surface of branched polyethylenimine to overcome the inclusion of the ligand into the interior of cyclodextrin. This study is an example of why it is essential to consider the interaction of components available on the surface of the nanoparticles to improve their effectiveness. The supramolecular interaction between the aromatic region of the folic acid and the hydrophobic interior of β-cyclodextrin limits the number of folic acids available to bind with the folate receptor, which is apparent from the data provided by the folate receptor-binding study. In a study by Díaz-García et al. (2020), it is demonstrated that the folate-functionalized mesoporous nanostructured silica systems used were more active in targeting FR-overexpressed cancer cells when the quantity of conjugated folate was higher. The IC_50_ value for MSN-AP-FA-Sn nanoparticles (with 10% folate) was higher than MSN-AP-FA25-Sn (with 25% folate) when tested against FR-overexpressed ovarian adenocarcinoma OVCAR-3 (IC_50_ value 50.36 ± 3.82 μg/mL versus 22.49 ± 1.43 μg/mL, respectively).

The key paper using folate-functionalized exosome for siRNA delivery aims to overcome endosomal trapping [30], which issues seem barely discussed in other literature harnessing folate targeting. Endosomal entrapment is an issue common to the drug delivery systems transported into the cells via endocytic mechanisms, subsequently, entering the slightly acidic early endosome. The vacuolar ATPase actively pumps protons into the endosome, making the endosome progressively mature into the late endosome, lowering the luminal pH to around 5.5. Then, the late endosome fuses with a lysosome, followed by the degradation of intraluminal contents by lysosomal enzymes. The drug delivery systems need to escape the endosomal/lysosomal prior to the degradation. However, most delivery systems reported a very inefficient endosomal escape process. Hence, most of the cargo was trapped inside the endosome/lysosome. There is a lack of comprehension of the endosomal escape mechanisms or drug delivery systems that productively overcome endosomal entrapment [33].

### 4.2. Concerns Regarding Folate-Functionalized Nanoparticles

In vivo biodistribution study is essential to determine whether the synthesized nanoparticles selectively target the tumor. The nanoparticle accumulation in normal tissues or organs may lead to unfavorable side effects. In a study using folate-targeted liposomes loaded with methotrexate to treat rheumatoid arthritis [17], the nanoparticles highly accumulated in the arthritic mice joints (male 6-week-old DBA/1 mice, vulnerable to collagen-induced arthritis). Interestingly, the liposomes were formulated using a folate-peptide, which claimed to be more efficient than the traditional system linking the folic acid to PEG in liposomes.

Nonetheless, there is no significant difference between the concentration of liposomes with or without the folate ligand in affected murine paws observed 72 h post injection using nuclear imaging. In contrast, the folate-targeted liposomes appeared to accumulate in the liver and spleen more rapidly, labeling both organs distinctively. The non-targeting liposomes’ signal increased more slowly in the spleen. The folate-targeting liposomes appeared to be concentrated to a significant extent in proximal lymph nodes in affected paws. No differences were observed between both liposomes’ concentration in the serum. A previously mentioned study using folate-functionalized PEG-modified PAMAM G4 dendrimers to target breast tumors observed a similar biodistribution behavior [24]. The percentages of injected radiolabeled nano complex (dose per gram of tumor/organ) after 24 h of intravenous administration were 13.76 ± 1.39% for tumor tissue, 13.62 ± 1.29% (liver), 1.54 ± 0.07% (lung), 4.45 ± 0.43% (kidney), and 3.28 ± 0.28% (spleen). A similar large portion of nanoparticles accumulated in both tumor tissue and liver 24 h post intravenous administration. It is essential to mention that the FR-overexpressed mouse breast carcinoma 4 T1 cell was established in the murine model by the subcutaneous injection on the right side of the post-neck region.

Another study using intravenously injected ^64^Cu-radiolabeled folate-targeting liposomes in six-week-old female, athymic nude mice, NMRI nu/nu observed a similar case [14]. The KB cancer cells were subcutaneously inoculated on both flanks after the mice were allowed to acclimatize for a week. The liver rapidly took up the folate-targeting liposomes compared to the non-targeting, reducing the circulating half-life of the liposomes. Subsequently, the overall delivery to the FR-overexpressed KB carcinoma xenografts showed no improvement. This study was based on previous research demonstrating improved therapeutic efficacy of encapsulated doxorubicin in folate-targeting liposomes compared to the non-targeting in small KB xenografts [34]. Perhaps the contradicting results observed might be due to the different formulations and experimental settings used. In the 2010 study, the KB tumor cells were inoculated in the right hind footpad of 8–10-week-old female nude mice.

In another study utilizing folate-functionalized PEG-coated liposomes to target FR-β positive macrophages in atherosclerosis mice [27], the biodistribution visualized using fluorescence demonstrated that the folate-functionalized liposomes primarily accumulated in the inflamed aorta/heart region up to 4 days post intraperitoneal administration. Then, the relative fluorescent intensity dropped from that region, followed by increasing fluorescent intensity in the liver region up to day 7.

Another study investigating intravenously administered folate-targeting ACGs’ nanosuspension against subcutaneously inoculated HeLa tumor-bearing female Balb/c nude mice reported significantly higher fluorescent intensity for both folate- and non-targeting nanosuspensions in the liver compared to the tumor [22]. However, the fluorescent intensity for folate-targeting in the liver and tumor was significantly higher than the non-targeting. Still, the folate-functionalized nanosuspensions showed better tumor growth inhibition than the counterpart, further supported by higher targeting efficiency for the folate-functionalized than the non-targeting. It is to note that the targeting efficiency was determined by the ratio of the fluorescent intensity in the tumor to the liver.

In another previously mentioned study using oleuropein-loaded, folate-modified PEG liposomes [25], it seemed the folate-targeting liposome had a unique pharmacokinetic profile compared to its non-targeting counterpart. The active targeting liposome concentration in the subcutaneously injected 22Rv1 prostate tumor is significantly higher than in other organs across the 48 h post intravenous administration. The peak concentration of non-targeting liposomes in the lungs is significantly higher than its counterpart, although the time taken to reach the peak is slower. However, it seemed the folate-targeting liposomes preferentially accumulated in the kidney more than its counterpart. The peak concentration for both liposomes seem relatively similar in the heart and spleen. The concentration for the non-targeting liposomes is slightly higher than its counterpart in the liver. A proper statistical analysis may help conclusively determine whether there is any significant difference between both liposomes’ pharmacokinetic profiles. Some possible explanations for the accumulation in other organs are long circulation period and enhanced permeation and retention effect (EPR). The overall data suggested no apparent problems with the organ accumulation on the targeting and anticancer effectiveness.

In another study [21], an in vivo uptake study using a polymeric drug delivery vehicle composed of folic acid, fluorescein isothiocyanate (FITC), and central PEG core for targeted hepatocellular carcinoma detection demonstrated that there is a distinct biodistribution between the intravenous and intra-arterial administration. The tumor xenograft was prepared by injecting N1S1 rat hepatoma directly into the exposed left hepatic lobe. The live animal imaging performed 30 min post treatment using the Sprague–Dawley HCC rat model found no potential FITC signal observed in the liver tumor region with intravenous administration. In contrast, the FITC signal of interest was detected with intra-arterial administration.

Furthermore, the study utilizing triblock copolymer delivering siRNA to the orthotopic ovarian murine model demonstrated different biodistribution profiles for the intravenous and intraperitoneal administrations [18]. The SKOV-3/LUC human ovary cancer/luciferase cell line was administered intraperitoneally to imitate the clinical manifestation of ovarian cancer. For the intravenous injection, a large portion of the injected dose accumulated in the liver, with the non-targeting nanoparticles accumulating higher than the targeting (53% versus 38% ID/g, respectively). Only a tiny amount of both nanoparticles accumulated in the tumor. In the case of intraperitoneal injection, there was a significant uptake by the kidneys for both formulations. The study suggested that kidney accumulation occurred mainly through passive renal accumulation possibly by the enhanced permeation and retention (EPR) effect, instead of active targeting due to the FR expressed within the proximal tubules being unavailable for access via the blood circulation. The rapid elimination from the circulation into the liver makes intravenous injection undesirable for folate-targeting nanoparticles.

It is well-established that most administered nanoparticles would not get to the desired target, and instead are sequestered by the spleen and liver. It may be recognized as foreign materials, triggering mononuclear phagocyte system uptake abounding in the spleen and liver [22]. The accumulation possibly causes increasing toxicity at the hepatic cellular level. One of the important macrophages present in the liver are Kupffer cells, involved in the first line of innate immunity. Kupffer cells phagocytose and destroy pathogens and other foreign objects present in the blood. The uptake and retention rate are highly associated with the nanoparticle’s surface charge, size, and ligand chemistry. In vitro study shows that nanoparticles with strong anionic or cationic surface charges will interact with serum protein, forming a protein corona and aggregates, causing an increased interaction with macrophages [35,36]. PEGylated nanoparticles are less efficiently taken by phagocytes. Sinusoidal endothelial cells found lining the vasculature of the liver sinusoid are also involved in innate immunity. It is involved in the elimination of waste macromolecules, including components of connective tissue and hyaluronan from blood circulation by receptor–ligand interactions. Both Kupffer cells and sinusoidal endothelial cells likely compete for the nanoparticles present in the circulation. In addition, the nanoparticles’ physicochemical properties, such as the hydrodynamic size, influence the mechanism of cellular internalization. Nanoparticles with a diameter greater than 100 nm or aggregated nanoparticles lower than 100 nm will have greater interaction with Kupffer cells. In contrast, sinusoidal endothelial cells might take up smaller monodisperse nanoparticles to a higher degree [36]. Several key studies discussed above found that the liver preferentially took up the folate-targeting nanoparticles more than its non-targeting counterpart. Perhaps this phenomenon might be due to the FR-β expressed by activated macrophages present in the liver. Several studies noted that the EPR effect may have a lack of impact on short-circulating folate-functionalized nanoparticles due to rapid uptake by the liver [14,18].

Altering folate blood serum levels may have an impact on the folate-functionalized nanoparticles’ effectiveness by affecting liver uptake. Low-folate diets are demonstrated to elevate tissue retention of the folate-functionalized carrier, subsequently depleting the amount present in the circulation [14,37]. A study demonstrated that pre-treatment with excess folic acid yielded no difference between folate-targeting and non-targeting liposomes and their tumor uptake levels, suggesting that folic acid competitively inhibits the interaction between the nanoparticles and FR. The preinjected folic acid only reduced the liver uptake for a short period, which may be insufficient for long-circulating liposomes. Multiple doses at intervals or sustained-release folic acid may help to reduce the liver uptake further, albeit it may affect the tumor uptake [14]. Perhaps, manipulating the preinjected folic acid doses to reduce liver uptake while minimally affecting tumor uptake may be possible; or, an inhibitor selectively targeting activated FR-β macrophages instead of FR-α tumor may be an option in the future. The crystallographic works by Wibowo et al. (2013) may catalyze the possibility of designing folate-targeting agents selective to either FR-α or FR-β for specific disease treatment. The study found that the binding of folates to both FR-α and FR-β are pH-dependent, while lesser changes for antifolates pemetrexed, aminopterin, and methotrexate [38]. Another compelling design strategy involves using a PEG-shielded surface capable of dePEGylation at the target site, exposing the masked targeting ligands to assist intracellular uptake. This strategy is plausible to show higher success than current strategies, most of which utilized targeting folate conjugated at the distal end of PEG moiety [39]. A study by McNeeley et al. (2009) successfully developed folate-functionalized liposomes with cysteine-cleavable PEG_5000_ coating designed to target brain tumors. Pre-clinical studies have demonstrated that the detachable PEG coating improved circulation time. The presence of cysteine at the targeted brain tumor induced the detachment of PEG, subsequently exposing the folic acid [39,40]. The summary of the problems discussed and their potential solutions are listed in Table 2 below.

## 5. Conclusions

Actively targeting folate receptors using a ligand is a promising strategy to improve therapeutic agents’ selectivity with several antibody–drug conjugates currently undergoing clinical trials. However, it seems like no folate-functionalized nanoparticle, particularly useful for conventional therapeutic payload with low tumor selectivity, successfully enter the clinical trials. Based on recently published pre-clinical data, there are abundant in vitro evidences for folate-targeting effectiveness compared to non-targeting counterparts and free drugs. An in vitro study emphasizes the importance of characterizing the nanoparticles’ surface properties to predict or optimize their targeting effectiveness, which is lacking in various literature. From the discussion above, PEG lengths critically affect the protein–surface interaction, which is perhaps the answer to why some folate-PEG nanoparticles reported have short circulating periods despite PEGylated being a known method to reduce phagocytic cells’ uptake. It is puzzling to generalize many nanoparticles reported due to different compositions, plus, lacking advanced characterization of the nanoparticles formulated makes it even harder. Despite numerous in vitro evidence to support the folate-targeting effectiveness, in vivo study is essential to understand the folate-targeting behavior in a living model, including limitations that may arise from it. Rapid liver accumulation is potentially causing renal toxicity and reducing tumor uptake, rendering the targeting ineffective. Hence, exploring solutions to this issue may help to improve folate-functionalized nanoparticles’ effectiveness in a living model. Additionally, it is logical to hypothesize that folate-targeting nanoparticles may be inefficient in cancer patients with inflammatory diseases, something worth exploring in the future. The route of administration might also affect the targeting. Hence, it is essential to consider the tumor’s location when determining the route of administration. Finally, it is worth mentioning that many reported in vivo models discussed above lack an actual representation of the cancer anatomy in clinical settings. Notably, most tumor xenografts are established by subcutaneous injection away from the orthotopic location of the tumor. Perhaps utilizing an orthotopic cancer model might further confirm the effectiveness of the folate-targeting nanoparticles. Optimistically, further insight into the factors affecting folate-functionalized nanoparticle drug delivery systems’ effectiveness and its potential concerns may advance its utility for patients’ treatment.

## Figures and Tables

**Table 2:** The summary of the problems discussed and their potential solutions.

Problems	Potential Solutions
Significant liver accumulation	Alternative administration route [18]. PEGylated nanoparticles are less efficiently taken by liver phagocytes. PEG-shielded surface capable of dePEGylation at the target site [38]. Multiple doses of pre-injected folic acid or sustained-release folic acid [14]. An inhibitor selectively targeting activated FR-β macrophages instead of FR-α tumor.
Formation of protein corona	Introducing (2-hydroxypropyl)-β-cyclodextrin (HPβCD) into the formulation to promote the PEG chain extension and folate exposition. The usage of shorter PEG length [28].
Endosomal trapping	More investigation is required to understand the endosomal escape mechanism [33].

## Data Availability

Not applicable.

## References

[1] Danhier F., Feron O., Préat V. (2010). To exploit the tumor microenvironment: Passive and active tumor targeting of nanocarriers for anti-cancer drug delivery. J. Control. Release.

[2] Rana A., Bhatnagar S. (2021). Advancements in folate receptor targeting for anti-cancer therapy: A small molecule-drug conjugate approach. Bioorg. Chem..

[3] Leamon C.P., Reddy J.A. (2004). Folate-targeted chemotherapy. Adv. Drug Deliv. Rev..

[4] Kamen B.A., Capdevila A. (1986). Receptor-mediated folate accumulation is regulated by the cellular folate content. Proc. Natl. Acad. Sci. USA.

[5] Cheng X., Li J., Tanaka K., Majumder U., Milinichik A.Z., Verdi A.C., Maddage C.J., Rybinski K.A., Fernando S., Fernando D. (2018). MORAb-202, an antibody–drug conjugate utilizing humanized anti-human FRa farletuzumab and the microtubule-targeting agent eribulin, has potent antitumor activity. Mol. Cancer Ther..

[6] Shimizu T., Fujiwara Y., Yonemori K., Koyama T., Sato J., Tamura K., Shimomura A., Ikezawa H., Nomoto M., Furuuchi K. (2021). First-in-human phase 1 study of MORAb-202, an antibody–Drug conjugate comprising farletuzumab linked to Eribulin Mesylate, in patients with folate receptor-a–Positive advanced solid tumors. Clin. Cancer Res..

[7] Moore K.N., Borghaei H., O’Malley D.M., Jeong W., Seward S.M., Bauer T.M., Perez R.P., Matulonis U.A., Running K.L., Zhang X. (2017). Phase 1 Dose-Escalation Study of Mirvetuximab Soravtansine (IMGN853), a Folate Receptor α-Targeting Antibody-Drug Conjugate, in Patients With Solid Tumors. Cancer.

[8] Moore K.N., Martin L.P., O’Malley D.M., Matulonis U.A., Konner J.A., Perez R.P., Bauer T.M., Ruiz-Soto R., Birrer M.J. (2017). Safety and activity of mirvetuximab soravtansine (IMGN853), a folate receptor alpha-targeting antibody-drug conjugate, in platinum-resistant ovarian, fallopian tube, or primary peritoneal cancer: A phase i expansion study. J. Clin. Oncol..

[9] O’Malley D.M., Matulonis U.A., Birrer M.J., Castro C.M., Gilbert L., Vergote I., Martin L.P., Mantia-Smaldone G.M., Martin A.G., Bratos R. (2020). Phase Ib study of mirvetuximab soravtansine, a folate receptor alpha (FRα)-targeting antibody-drug conjugate (ADC), in combination with bevacizumab in patients with platinum-resistant ovarian cancer. Gynecol. Oncol..

[10] Moore K.N., O’Malley D.M., Vergote I., Martin L.P., Gonzalez-Martin A., Malek K., Birrer M.J. (2018). Safety and activity findings from a phase 1b escalation study of mirvetuximab soravtansine, a folate receptor alpha (FRα)-targeting antibody-drug conjugate (ADC), in combination with carboplatin in patients with platinum-sensitive ovarian cancer. Gynecol. Oncol..

[11] Buskaran K., Bullo S., Hussein M.Z., Masarudin M.J., Mohd Moklas M.A., Fakurazi S. (2021). Anticancer molecular mechanism of protocatechuic acid loaded on folate coated functionalized graphene oxide nanocomposite delivery system in human hepatocellular carcinoma. Materials.

[12] Buskaran K., Hussein M.Z., Moklas M.A.M., Masarudin M.J., Fakurazi S. (2021). Graphene oxide loaded with protocatechuic acid and chlorogenic acid dual drug nanodelivery system for human hepatocellular carcinoma therapeutic application. Int. J. Mol. Sci..

[13] Charbgoo F., Nikkhah M., Behmanesh M. (2018). Size of single-wall carbon nanotube affects the folate receptor-mediated cancer cell targeting. Biotechnol. Appl. Biochem..

[14] Christensen E., Henriksen J.R., Jørgensen J.T., Amitay Y., Shmeeda H., Gabizon A.A., Kjær A., Andresen T.L., Hansen A.E. (2018). Folate receptor targeting of radiolabeled liposomes reduces intratumoral liposome accumulation in human KB carcinoma xenografts. Int. J. Nanomed..

[15] Díaz-García D., Montalbán-Hernández K., Mena-Palomo I., Achimas-Cadariu P., Rodríguez-Diéguez A., López-Collazo E., Prashar S., Paredes K.O., Filice M., Fischer-Fodor E. (2020). Role of folic acid in the therapeutic action of nanostructured porous silica functionalized with organotin(IV) compounds against different cancer cell lines. Pharmaceutics.

[16] El-Far S.W., Helmy M.W., Khattab S.N., Bekhit A.A., Hussein A.A., Elzoghby A.O. (2018). Folate conjugated vs PEGylated phytosomal casein nanocarriers for codelivery of fungal- and herbal-derived anticancer drugs. Nanomedicine.

[17] Guimarães D., Lager F., Renault G., Guezguez J., Burnet M., Cunha J., Cavaco-Paulo A., Nogueira E. (2022). Folate-Targeted Liposomal Formulations Improve Effects of Methotrexate in Murine Collagen-Induced Arthritis. Biomedicines.

[18] Jones S.K., Douglas K., Shields A.F., Merkel O.M. (2018). Correlating quantitative tumor accumulation and gene knockdown using SPECT/CT and bioluminescence imaging within an orthotopic ovarian cancer model. Biomaterials.

[19] Kasprzak A., Grudzinski I.P., Bamburowicz-Klimkowska M., Parzonko A., Gawlak M., Poplawska M. (2018). New Insight into the Synthesis and Biological Activity of the Polymeric Materials Consisting of Folic Acid and β-Cyclodextrin. Macromol. Biosci..

[20] Khan M.M., Madni A., Filipczak N., Pan J., Rehman M., Rai N., Attia S.A., Torchilin V.P. (2020). Folate targeted lipid chitosan hybrid nanoparticles for enhanced anti-tumor efficacy. Nanomed. Nanotechnol. Biol. Med..

[21] Koirala N., Das D., Fayazzadeh E., Sen S., McClain A., Puskas J.E., Drazba J.A., McLennan G. (2019). Folic acid conjugated polymeric drug delivery vehicle for targeted cancer detection in hepatocellular carcinoma. J. Biomed. Mater. Res. Part A.

[22] Li H., Li Y., Ao H., Bi D., Han M., Guo Y., Wang X. (2018). Folate-targeting annonaceous acetogenins nanosuspensions: Significantly enhanced antitumor efficacy in hela tumor-bearing mice. Drug Deliv..

[23] Maiyo F., Singh M. (2019). Folate-targeted mRNA delivery using chitosan- functionalized selenium nanoparticles: Potential in cancer immunotherapy. Pharmaceuticals.

[24] Narmani A., Arani M.A.A., Mohammadnejad J., Vaziri A.Z., Solymani S., Yavari K., Talebi F., Darzi S.J. (2020). Breast Tumor Targeting with PAMAM-PEG-5FU-^99m^Tc as a New Therapeutic Nanocomplex: In In-vitro and In-vivo studies. Biomed. Microdevices.

[25] Nassir A.M., Ibrahim I.A.A., Md S., Waris M., Tanuja Ain M.R., Ahmad I., Shahzad N. (2019). Surface functionalized folate targeted oleuropein nano-liposomes for prostate tumor targeting: In vitro and in vivo activity. Life Sci..

[26] Pawar A., Singh S., Rajalakshmi S., Shaikh K., Bothiraja C. (2018). Development of fisetin-loaded folate functionalized pluronic micelles for breast cancer targeting. Artif. Cells Nanomed. Biotechnol..

[27] Poh S., Chelvam V., Ayala-López W., Putt K.S., Low P.S. (2018). Selective liposome targeting of folate receptor positive immune cells in inflammatory diseases. Nanomed. Nanotechnol. Biol. Med..

[28] Venuta A., Moret F., Poggetto G.D., Esposito D., Fraix A., Avitabile C., Ungaro F., Malinconico M., Sortino S., Romanelli A. (2018). Shedding light on surface exposition of poly(ethylene glycol) and folate targeting units on nanoparticles of poly(ε-caprolactone) diblock copolymers: Beyond a paradigm. Eur. J. Pharm. Sci..

[29] Xu L., Jiang G., Chen H., Zan Y., Hong S., Zhang T., Zhang Y., Pei R. (2019). Folic acid-modified fluorescent dye-protein nanoparticles for the targeted tumor cell imaging. Talanta.

[30] Zheng Z., Li Z., Xu C., Guo B., Guo P. (2019). Folate-displaying exosome mediated cytosolic delivery of siRNA avoiding endosome trapping. J. Control. Release.

[31] Zou Y., Xiao F., Song L., Sun B., Sun D., Chu D., Wang L., Han S., Yu Z., O’Driscoll C.M. (2021). A folate-targeted PEGylated cyclodextrin-based nanoformulation achieves co-delivery of docetaxel and siRNA for colorectal cancer. Int. J. Pharm..

[32] Conte C., Fotticchia I., Tirino P., Moret F., Pagano B., Gref R., Ungaro F., Reddi E., Giancola C., Quaglia F. (2016). Cyclodextrin-assisted assembly of PEGylated polyester nanoparticles decorated with folate. Colloids Surf. B Biointerfaces.

[33] Pei D., Buyanova M. (2019). Overcoming Endosomal Entrapment in Drug Delivery. Bioconjug. Chem..

[34] Gabizon A., Tzemach D., Gorin J., Mak L., Amitay Y., Shmeeda H., Zalipsky S. (2010). Improved therapeutic activity of folate-targeted liposomal doxorubicin in folate receptor-expressing tumor models. Cancer Chemother. Pharmacol..

[35] Walkey C.D., Olsen J.B., Guo H., Emili A., Chan W.C.W. (2012). Nanoparticle Size and Surface Chemistry Determine Serum Protein Adsorption and Macrophage Uptake. J. Am. Chem. Soc..

[36] Zhang Y.N., Poon W., Tavares A.J., McGilvray I.D., Chan W.C.W. (2016). Nanoparticle–liver interactions: Cellular uptake and hepatobiliary elimination. J. Control. Release.

[37] Leamon C.P., Reddy J.A., Dorton R., Bloomfield A., Emsweller K., Parker N., Westrick E. (2008). Impact of high and low folate diets on tissue folate receptor levels and antitumor responses toward folate-drug conjugates. J. Pharmacol. Exp. Ther..

[38] Wibowo A.S., Singh M., Reeder K.M., Carter J.J., Kovach A.R., Meng W., Ratnam M., Zhang F., Dann C.E. (2013). Structures of human folate receptors reveal biological trafficking states and diversity in folate and antifolate recognition. Proc. Natl. Acad. Sci. USA.

[39] Yoo J.W., Doshi N., Mitragotri S. (2011). Adaptive micro and nanoparticles: Temporal control over carrier properties to facilitate drug delivery. Adv. Drug Deliv. Rev..

[40] McNeeley K.M., Karathanasis E., Annapragada A.V., Bellamkonda R.V. (2009). Masking and triggered unmasking of targeting ligands on nanocarriers to improve drug delivery to brain tumors. Biomaterials.

